# Force to Rebalance Control of HRG and Suppression of Its Errors on the Basis of FPGA

**DOI:** 10.3390/s111211761

**Published:** 2011-12-16

**Authors:** Xu Wang, Wenqi Wu, Bing Luo, Zhen Fang, Yun Li, Qingan Jiang

**Affiliations:** 1 College of Mechanical Engineering and Automation, National University of Defense Technology, Changsha 410073, Hunan, China; E-Mails: wenqiwu_lit@hotmail.com (W.W.); ruobing@nudt.edu.cn (B.L.); ly20090801@163.com (Y.L.); jqa1987@163.com(Q.J.); 2 Institution of Piezoelectric and Acousto-Optic Technology, Chongqing 400060, China; E-Mail: hrg@sipat.com

**Keywords:** Hemispherical Resonator Gyro (HRG), force-to-rebalance control, FPGA, quadrature error, rate sensor

## Abstract

A novel design of force to rebalance control for a hemispherical resonator gyro (HRG) based on FPGA is demonstrated in this paper. The proposed design takes advantage of the automatic gain control loop and phase lock loop configuration in the drive mode while making full use of the quadrature control loop and rebalance control loop in controlling the oscillating dynamics in the sense mode. First, the math model of HRG with inhomogeneous damping and frequency split is theoretically analyzed. In addition, the major drift mechanisms in the HRG are described and the methods that can suppress the gyro drift are mentioned. Based on the math model and drift mechanisms suppression method, four control loops are employed to realize the manipulation of the HRG by using a FPGA circuit. The reference-phase loop and amplitude control loop are used to maintain the vibration of primary mode at its natural frequency with constant amplitude. The frequency split is readily eliminated by the quadrature loop with a DC voltage feedback from the quadrature component of the node. The secondary mode response to the angle rate input is nullified by the rebalance control loop. In order to validate the effect of the digital control of HRG, experiments are carried out with a turntable. The experimental results show that the design is suitable for the control of HRG which has good linearity scale factor and bias stability.

## 1. Introduction

A hemispherical resonator gyro is a solid state gyroscope which has achieved inertial grade performance. It has the features of high accuracy, long life span, inherent high reliability and no wear-out parts. With its excellent performance, the HRG can be used for spacecraft and satellite stabilization, precision pointing, aircraft navigation, strategic accuracy systems, oil borehole exploration, as well as planetary exploration [1,2].

Owing to its great number of advantages, many researchers are devoted to research related to it. Loper and Lynch at General Motors were the first researchers who described the HRG as a rate integrating gyroscope, which is operated under a “Whole Angle” (WA) mode situation [3,4]. In [4], the principle of WA mode operation is reviewed and its electrical control and readout methods are also discussed in detail. In this mode, the standing wave of a vibrating pattern is allowed to precess freely around the circumference of the resonator. This “whole angle” mode has the unique advantage that the device would continue to integrate the applied rotation during short electrical power interruptions so that it could endure nuclear explosions [5]. Later on, Matthews employed the HRG with a 30 mm in diameter resonator operating as a rate gyroscope, which is operated under the condition of “Force-To-Rebalance” (FTR) mode [6,7]. In this mode, the standing wave of the vibrating pattern is maintained at a prescribed position, usually with the antinode of the standing wave aligning with 0° electrode. In addition, the input angle rate information is related to the feedback voltage of the rebalance control loop which is used to make the standing waves align with the special electrode. Comparing the two working modes, the “Force-To-Rebalance” mode has a higher resolution and a much simpler readout system than that of the “Whole Angle” mode [8].

Since any imperfection of the resonator would result in many errors, a lot of papers have concentrated on the topic of error reduction of the gyro. Lynch introduced an averaging method to develop a math model of the HRG and analyzed the main errors which may give rise to drift of the gyro [4]. Matthews briefly introduced the design and operation of the HRG, and described the modifications made to achieve a very low noise and high resolution of HRG for precision spacecraft pointing [9,10]. Based on his modifications, the HRG offers precision performance through electronics design improvement without any changes to the HRG instrument. The resonator has various Q-factors, so the damping constants of the standing wave depend on its orientation. Zhbanov studied the methods to decrease the level of dynamic errors of the amplitude maintenance contour, and simultaneously eliminate the drift caused by differences in Q-factors [11]. In [12], he also studied the effect of movability of the resonator center on the operation of HRG. In [13,14], Shatalov analyzed the influence of mass and damping imperfections on the evolution of standing waves in the HRG resonator. Moreover, he offered proposal for compensating those drifts by means of special control over the vibrating pattern by a sectioned parametric electrode. In [5,15,16], Loveday employed a control system to reduce the effects of imperfections in a cylinder vibrating gyro, a method that could shed some light on the study of HRG.

These studies mainly focus on the effects of imperfections on the resonator dynamics and the improvement of HRG operating performance. However, the design of the control systems for HRG has not been well documented in the literature and also the embodiments of the control loop which ensure the HRG to achieve high performances have only received limited attention. Moreover, studies put much emphasis on the WA mode while those on the FTR mode are rare. In this paper, theoretical and experimental studies on the characterization of a HRG are presented. Both the embodiments of the control loops which realize control algorithm and the experimental data of the gyro are provided under the FTR mode.

## 2. General Model of HRG

**Figure 1.**
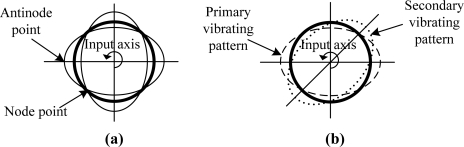
**(a)** Standing wave on the resonator with four nodes and antinodes. **(b)** Primary vibrating pattern and secondary vibrating pattern.

A standing wave, which has four nodes and antinodes, as shown in Figure 1(a), can be sustained in the resonator by the driving of the electrodes. When the resonator is rotated, energy is coupled from primary vibration mode into a secondary vibration mode. The inertial rotation can be thought of as causing the amplitude of the primary vibration pattern to decrease and a new component pattern (secondary vibrating pattern) to build up whose antinodes lie 45° from the original pattern antinodes; that is, whose antinodes coincide with the nodes of the original pattern as shown in Figure 1(b). The superposition of these two pattern components produces the resultant pattern [4]. For a perfect resonator, there are two modes of equal frequency and identical mode shape, circumferentially spaced relative to each other at 45° [5]. The magnitude of the response of the secondary mode is then proportional to the applied rate of turn. In FTR mode, the response of the secondary mode is nulled by the rebalance control loop instantaneously. Consequently, the rotation rate can be obtained from the force which is used for nulling the response of the second mode.

The math model proposed by Lynch is used in this paper with the resonator operating in the n = 2 mode [17,18]. In the math model, mass and stiffness imperfections causing frequency split between the two modes and inhomogeneous damping dependent on the circumference angle are considered. The equations are presented as follows:

(1) $$\{\begin{array}{l} \ddot{x}-2\mathrm{nk}\Omega \dot{y}+\left(\frac{2}{\tau }+\Delta \left(\frac{1}{\tau }\right)\;\text{cos}\;2n\theta_{\tau }\right)\dot{x}+\Delta \left(\frac{1}{\tau }\right)\;\text{sin}\;2n\theta_{\tau }\dot{y}+\left(\omega^{2}-\omega \Delta \omega \;\text{cos}\;2n\theta_{\omega }\right)x-\omega \Delta \omega \;\text{sin}\;2n\theta_{\omega }y=f_{x}\\ \ddot{y}+2\mathrm{nk}\Omega \dot{x}+\left(\frac{2}{\tau }-\Delta \left(\frac{1}{\tau }\right)\;\text{cos}\;2n\theta_{\tau }\right)\dot{y}+\Delta \left(\frac{1}{\tau }\right)\;\text{sin}\;2n\theta_{\tau }\dot{x}+\left(\omega^{2}-\omega \Delta \omega \;\text{cos}\;2n\theta_{\omega }\right)y-\omega \Delta \omega \;\text{sin}\;2n\theta_{\omega }x=f_{y} \end{array}$$

where:

$$\omega^{2}=\frac{\omega_{x}^{2}+\omega_{y}^{2}}{2},\omega \Delta \omega =\frac{\omega_{x}^{2}-\omega_{y}^{2}}{2},\frac{1}{\tau }=\frac{1}{2}\left(\frac{1}{\tau_{x}}+\frac{1}{\tau_{y}}\right),\Delta \left(\frac{1}{\tau }\right)=\left(\frac{1}{\tau_{x}}-\frac{1}{\tau_{y}}\right)$$

The Equation (1) can be rewritten as the following form:

(2) $$\{\begin{array}{l} \ddot{x}-2\mathrm{nk}\Omega \dot{y}+D_{\mathrm{xx}}\dot{x}+D_{\mathrm{xy}}\dot{y}+k_{\mathrm{xx}}x+k_{\mathrm{xy}}y=f_{x}\\ \ddot{y}+2\mathrm{nk}\Omega \dot{x}+D_{\mathrm{yx}}\dot{x}+D_{\mathrm{yy}}\dot{y}+k_{\mathrm{yx}}x+k_{\mathrm{yy}}y=f_{y} \end{array}$$

where:

$$\begin{array}{c} D_{\mathrm{xx}}=\frac{2}{\tau }+\Delta \left(\frac{1}{\tau }\right)\;\text{cos}\;2n\theta_{\tau },k_{\mathrm{xx}}=\omega^{2}-\omega \Delta \omega \;\text{cos}\;2n\theta_{\omega },D_{\mathrm{yy}}=\frac{2}{\tau }-\Delta \left(\frac{1}{\tau }\right)\;\text{cos}\;2n\theta_{\tau }\\ k_{\mathrm{yy}}=\omega^{2}+\omega \Delta \omega \;\text{cos}\;2n\theta_{\omega },D_{\mathrm{xy}}=D_{\mathrm{yx}}=\Delta \left(\frac{1}{\tau }\right)\;\text{sin}\;2n\theta_{\tau },k_{\mathrm{xy}}=k_{\mathrm{yx}}=\omega \Delta \omega \;\text{sin}\;2n\theta_{\omega } \end{array}$$

The x-directional resonator (primary mode) is typically excited at its resonance frequency *ω_x_* and its vibration amplitude is kept constant by the control force *f_x_*. Thus, the position of the x-direction vibrating can be written as:

(3) $$x=A\;\text{sin}\;\left(\omega_{x}t\right)$$

where *A* is the amplitude of the primary mode; *ω_x_* is x-direction resonance frequency. With a view to analyzing simpler, we assume that the driving force *f_y_* = 0, then, based on Equation (3), the equation for the secondary resonator becomes:

(4) $$\ddot{y}+D_{\mathrm{yy}}\dot{y}+k_{\mathrm{yy}}y=-\left(2\mathrm{nk}\Omega +D_{\mathrm{yx}}\right)A\omega_{x}\;\text{cos}\;\omega_{x}t-k_{\mathrm{yx}}A\;\text{sin}\;\omega_{x}t$$

The steady-state solution of Equation (4) can be written as:

(5) $$\begin{array}{l} y\left(t\right)=C_{1}\;\text{cos}\;\omega_{x}t+C_{2}\;\text{sin}\;\omega_{x}t\\ \;\;\;\;\;\;\;\;\;\;=\frac{\left(\omega_{x}^{2}-k_{\mathrm{yy}}\right)\left(2\mathrm{nk}\Omega +D_{\mathrm{yx}}\right)\omega_{x}+k_{\mathrm{yx}}D_{\mathrm{yy}}\omega_{x}}{{\left(\omega_{x}^{2}-k_{\mathrm{yy}}\right)}^{2}+{\left(D_{\mathrm{yy}}\omega_{x}\right)}^{2}}\\ \;\;\;\;\;\;\;\;\;\;+\frac{-\left(2\mathrm{nk}\Omega +D_{\mathrm{yx}}\right)D_{\mathrm{yy}}\omega_{x}^{2}-k_{\mathrm{yx}}\left(k_{\mathrm{yy}}-\omega_{x}^{2}\right)}{{\left(\omega_{x}^{2}-k_{\mathrm{yy}}\right)}^{2}+{\left(D_{\mathrm{yy}}\omega_{x}\right)}^{2}}A\;\text{sin}\;\omega_{x}t \end{array}$$

If the resonator is perfect with no frequency split and inhomogeneous damping, that is *ω_x_* = *ω_y_*, *k_yx_* = 0, $\Delta \left(\frac{1}{\tau }\right)=0$, *D_yx_* = 0, the Equation (5) will be simplified to:

(6) $$y\left(t\right)=-\frac{2\mathrm{nk}\Omega }{D_{\mathrm{yy}}}A\;\text{sin}\;\omega_{x}t$$

From Equation (6) we can see that in ideal HRG, the amplitude of the secondary mode is directly proportional to the input angle rate Ω, and the response is in-phase with the motion of the drive mode. In the FTR mode, the force rebalance control loop is employed to null this response, and therefore the applied rotation rate can be directly demodulated from the executing force that nullifies the response of secondary mode.

In practice, the perfect resonator cannot be fabricated, so some errors in the equation cannot be avoided. Let’s first take the inhomogeneous damping of the resonator into consideration, assuming that the primary mode and the second mode have the same natural frequency, which are Δ*ω* = 0, $\Delta \left(\frac{1}{\tau }\right)\neq 0$. Then Equation (5) becomes:

(7) $$y\left(t\right)=-\frac{2\mathrm{nk}\left(\Omega +\frac{D_{\mathrm{yx}}}{2\mathrm{nk}}\right)}{D_{\mathrm{yy}}}A\;\text{sin}\;\omega_{x}t$$

Equations (6) and (7) show that the inhomogeneous damping gives rise to an additional output signal corresponding to the angular rate which is absent in a perfect resonator. The additional output signal is:

(8) $$\Omega_{D_{\mathrm{yx}}}=\frac{D_{\mathrm{yx}}}{2\mathrm{nk}}$$

which is in phase with the actual Coriolis signal. In Equation (7) the Coriolis force is the only component we are interested in. The ideal situation for demodulation is that the false signal Ω*_D_yx__*, which will bring the gyroscope with zero-rate-output, is removed from the output of the gyroscope. But actually it is difficult to separate the false signal Ω*_D_yx__* from the truly useful signal Ω since they are in phase with each other and display the same effect. For this reason, in order to avoid deteriorating the performance of the gyroscope, the method of calibration should be employed to compensate the in phase error Ω*_D_yx__*.

Furthermore, we will continue to analyze the role that the frequency split plays on the response of the secondary mode, without considering the inhomogeneous damping around the circumference angle. Then the resonator is found to have two particular natural frequencies, which are *ω_x_* ≠ *ω_y_*, $\Delta \left(\frac{1}{\tau }\right)=0$, and the Equation (5) becomes:

(9) $$\begin{array}{l} y\left(t\right)=\frac{\left(\omega_{x}^{2}-k_{\mathrm{yy}}\right)2\mathrm{nk}\Omega \omega_{x}+k_{\mathrm{yx}}D_{\mathrm{yy}}\omega_{x}}{{\left(\omega_{x}^{2}-k_{\mathrm{yy}}\right)}^{2}+{\left(D_{\mathrm{yy}}\omega_{x}\right)}^{2}}A\;\text{cos}\;\omega_{x}t+\frac{-2\mathrm{nk}D_{\mathrm{yy}}\omega_{x}^{2}\Omega -k_{\mathrm{yx}}\left(k_{\mathrm{yy}}-\omega_{x}^{2}\right)}{{\left(\omega_{x}^{2}-k_{\mathrm{yy}}\right)}^{2}+{\left(D_{\mathrm{yy}}\omega_{x}\right)}^{2}}A\;\sin\;\omega_{x}t\\ \;\;\;\;\;\;\;\;\;=\frac{4\mathrm{nk}\Omega {\text{sin}}^{2}n\theta_{\omega }+\;\text{sin}\;2n\theta_{\omega }D_{\mathrm{yy}}}{{\left(2\omega \Delta \omega {\text{sin}}^{2}n\theta_{\omega }\right)}^{2}+{\left(D_{\mathrm{yy}}\omega_{x}\right)}^{2}}\omega_{x}\omega \Delta \omega A\;\text{cos}\;\omega_{x}t-\frac{2\mathrm{nk}D_{\mathrm{yy}}\omega_{x}^{2}\Omega -2k_{\mathrm{yx}}\omega \Delta {\text{sin}}^{2}n\theta_{\omega }}{{\left(2\omega \Delta \omega {\text{sin}}^{2}n\theta_{\omega }\right)}^{2}+{\left(D_{\mathrm{yy}}\omega_{x}\right)}^{2}}A\;\sin\;\omega_{x}t \end{array}$$

Comparison between Equations (6) and (9) shows that the presence of frequency split of the resonator has three effects on the output of the HRG. Firstly, it introduces an undesirable quadrature component which has a 90° phase shift related to the useful Coriolis signal. The Quadrature error term is shown as follows:

(10) $${\mathrm{Error}}_{\mathrm{quad}}=\frac{4\mathrm{nk}\Omega {\text{sin}}^{2}n\theta_{\omega }+\;\text{sin}\;2n\theta_{\omega }D_{\mathrm{yy}}}{{\left(2\omega \Delta \omega {\text{sin}}^{2}n\theta_{\omega }\right)}^{2}+{\left(D_{\mathrm{yy}}\omega_{x}\right)}^{2}}\omega_{x}\omega \Delta \omega A\;\text{cos}\;\omega_{x}t$$

**Figure 2.**
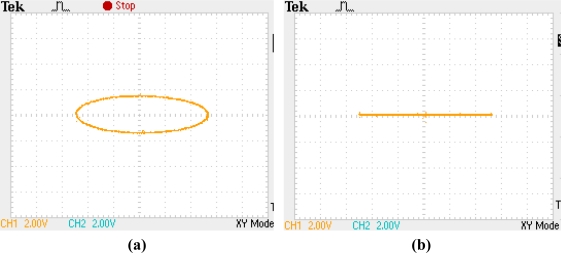
Lissajous figure on two electrodes with an angle distance by 45°. **(a)** An ellipse existing quadrature error. **(b)** A straight with the quadrature error suppressed.

Due to the quadrature error, the Lissajous figure on two electrodes with an angle distance by 45° will be an ellipse instead of a straight line as shown in Figure 2.

Secondly, with the term ${\left(\omega_{x}^{2}-k_{\mathrm{yy}}\right)}^{2}>0$, it greatly decreases the scale factor of the output of the gyro, which will consequently reduce the sensitivity of the HRG.

Thirdly, it is evident that the angular velocity information includes an error signal that is in phase with the Coriolis signal due to frequency split, and this error will bring the gyroscope with another zero-rate-output. The in-phase output of HRG is:

(11) $$-\frac{2\mathrm{nk}D_{\mathrm{yy}}\omega_{x}^{2}\Omega -2k_{\mathrm{yx}}\omega \Delta \omega {\text{sin}}^{2}n\theta_{\omega }}{{\left(2\omega \Delta \omega {\text{sin}}^{2}n\theta_{\omega }\right)}^{2}+{\left(D_{\mathrm{yy}}\omega_{x}\right)}^{2}}A\;\sin\;\omega_{x}t=-\frac{2\mathrm{nk}D_{\mathrm{yy}}\omega_{x}^{2}\left(\Omega -\frac{{\left(\omega \Delta \omega \right)}^{2}}{\mathrm{nk}D_{\mathrm{yy}}\omega_{x}^{2}}\text{sin}\;2n\theta_{\omega }{\text{sin}}^{2}n\theta_{\omega }\right)}{{\left(2\omega \Delta \omega {\text{sin}}^{2}n\theta_{\omega }\right)}^{2}+{\left(D_{\mathrm{yy}}\omega_{x}\right)}^{2}}A\;\sin\;\omega_{x}t$$

The additional in-phase component caused by frequency split is:

(12) $$\Omega_{k_{\mathrm{yx}}}=-\frac{{\left(\omega \Delta \omega \right)}^{2}}{\mathrm{nk}D_{\mathrm{yy}}\omega_{x}^{2}}\text{sin}\;2n\theta_{\omega }\;{\text{sin}}^{2}n\theta_{\omega }$$

This in phase term which is due to frequency split will be difficult to eliminate from the useful Coriolis force without considering other factors. However, since a frequency split will result in a quadrature error which could be separately observed, its feedback to eliminate the frequency split can finally eliminate the in phase error term brought about by the frequency split.

Based on the analysis, it can be observed that the output of gyro contains in phase error and quadrature error, both of which are partially caused at the same time by the frequency split. Therefore, only if the frequency split of the resonator is eliminated, can the performance of the gyro be improved. Additionally, the in phase error resulted from the inhomogeneous damping which causes zero-rate-output can be suppressed by the method of calibration. A high performance HRG will be achieved after taking the following factors into account:

1. Control the primary vibration precisely, which demands that the primary mode control should maintain the resonator at its natural frequency and at constant amplitude in spite of the changing environment.
2. Eliminate the frequency split so as to eliminate the in phase error Ω*_k_yx__* and quadrature error *Error_quad_* caused by it. While eliminating the frequency split, the Lissajous figure is a straight line, which will precess under the condition of an angle rate input. In the FTR mode, the feedback of rebalance force will sustain the line align with 0° electrode, from which the rotation angle rate can be demodulated.
3. In phase error caused by the inhomogeneous damping cannot be easily eliminated but can be compensated by method of calibration in advance.

## 3. Control of the HRG Under FTR Mode

Under FTR mode, in order to obtain a high performance of the HRG, four control loops are employed, namely, reference-phase loop, amplitude-control loop, quadrature-control loop and rebalance control loop [17]. The four loops are divided into two kinds; the first kind is used to control the primary vibration pattern while the second one is used to control the secondary vibration pattern.

### 3.1. Primary Vibration Pattern Design and Control [19]

**Figure 3.**
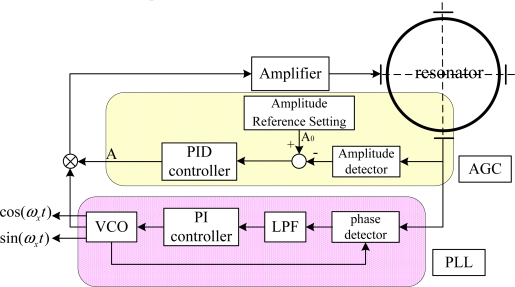
Primary control loops of the HRG including Phase-lock Loop (PLL) and Automatic Gain Control Loop (AGC).

The primary vibration pattern should be excited at its resonance frequency and the vibration amplitude should be kept constant at *A*_0_ by the control loop. However, both the amplitude and resonance frequency will vary due to environmental changes, such as changes in temperature or stiffness aging [20]. Therefore, reference-phase loop and amplitude-control loop are employed to control the variation of primary vibration pattern, which should be maintained at its natural frequency and at constant amplitude, as shown in Figure 3. A phase-locked loop (PLL) is used to realize the reference-phase loop function which tracks resonance frequency changes and tunes it to the resonance frequency. Meanwhile, an automatic gain control (AGC) loop is used to realize the amplitude-control loop function which maintains the amplitude constant, since constant amplitude of the primary vibration mode acts a decisive role in keeping the scale factor of the gyro constant.

#### 3.1.1. Reference-Phase Loop

In the resonator’s primary vibrating, frequency control is needed to track the change of resonant frequency due to environmental change and to tune it to a specified frequency vicinity of the natural frequency while having an appropriate phase. PLL is employed to realize this function. As shown in Figure 3, A PLL is composed of a phase detector, a low-pass filter (LPF), a PI controller, and a voltage-controlled oscillator (VCO). The antinodes vibrating signal are sent to the phase detector to compare with the signal from the VCO, and then the result of comparison passes through a low-pass filter and forms an error signal to control the VCO. This circuit is well justified by the experiment in the next section. Additionally, the PLL both provides the signals from which driving forces of the correct frequency and phase are derived and provides the reference signals for demodulating the output signals from the HRG.

The natural frequency of resonator will change as the temperature changes, so another important function of the PLL is for thermal modeling since the oscillation of the primary mode is locked to the natural frequency, whose frequency provides a direct measure of the temperature of the resonator [6,21].

#### 3.1.2. Amplitude Control Loop

The amplitude control is usually accomplished by ring electrode in WA mode, while completed by discrete electrode in FTR mode. The amplitude of the primary vibration will diminish due to damping, so force must be applied to sustain the amplitude against the damping losses. More importantly, the amplitude needs to maintain at its prescribed value regardless of the environmental change, and thus we employ an AGC loop in FTR mode, which is composed of an amplitude detector, a comparator and a PID controller. The antinodes vibrating signal is sent to the amplitude detector so as to obtain the amplitude, which is then compared to the prescribed value to get an error signal, from which the PID controller offers a correct driving force amplitude. It is of great necessity to maintain the constant amplitude of the primary vibration mode in order to keep the scale factor of the gyro constant.

### 3.2. Secondary Vibration Pattern Design and Control

As mentioned above, mechanical imperfections occur during the manufacture of the resonator, so it is inevitable to introduce some errors that seriously reduce the performance of the gyro. Among those errors, the mass unbalance, the frequency split and inhomogeneous damping are the main factors that contribute most to the drift of the gyro.

The mass unbalance imperfections cause the two vibration modes to have slightly different natural frequencies and determine the location of the mode shapes with respect to the structure [22,23]. The effect of mass unbalance imperfection can be minimized by an ion beam balancing procedure, which gets rid of unwanted mass on special distribution of the resonator. However, as this process is performed once at a single temperature, changes in the dynamics of the resonator over time or with temperature will not be accounted for [15]. To limit aerodynamic damping of the resonator, the resonator is enclosed in a vacuum. But usually resonator balancing procedure is performed at atmospheric pressures [16].

**Figure 4.**
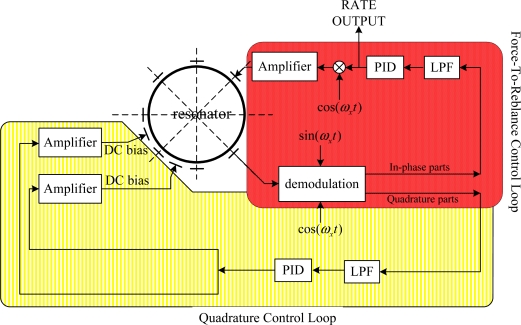
Secondary control loops of the HRG including Quadrature Control Loop and Rebalance Control Loop.

Therefore, the quadrature control loop which adopts a novelty feedback imposing a DC voltage on the electrodes to change the stiffness of the resonator should be employed to eliminate the frequency split of the two vibration modes. Simultaneously, the rebalance loop should also be employed to null the response of the second mode as illustrated in Figure 4. As a result, it looks as if the vibrating pattern is binding with the resonator while the procession effect disappears.

#### 3.2.1. Quadrature Control Loop

As discussed earlier, mass and stiffness imperfections cause frequency differences between the two modes. The effective stiffness of the resonator can be altered by electrostatic field produced by a DC voltage imposing on the electrodes since its effect can be represented as a negative spring. Consequently, the frequency split can be eliminated by the quadrature control loop which imposes a DC signal to the special electrodes through quadrature error feedback. As shown in Figure 4, the node signal is demodulated into two parts: in-phase part and quadrature part. The quadrature part which feeds back into the quadrature control loop is used as an error signal that produces a proportional DC signal to eliminate frequency split.

#### 3.2.2. Rebalance Control Loop

In FTR mode, the azimuth orientation of antinode is maintained at a prescribed position with respect to the resonator by the rebalance control loop. The prescribed position is usually set at the 0° electrode. The rebalance control loop acquires in-phase signal of the node points and the nodal motion is driven to a null. The voltage signals required to accomplish this are proportional to the rate at which the resonator is rotated around its symmetry axis [6].

## 4. Experiments

**Figure 5.**
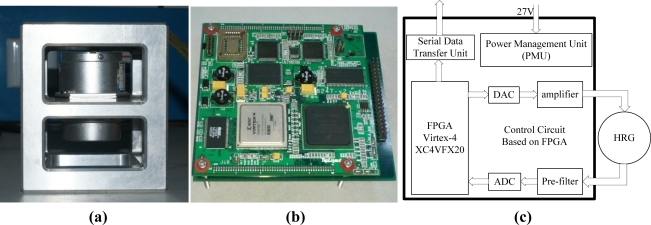
**(a)** The gauge outfit of HRG. **(b)** The PCB circuitry of HRG. **(c)** The overall construction of the circuit.

A PCB circuit board was designed based on the concepts shown in Figures 3 and 4. It employs a FPGA of the Virtex-4 XC4VFX20 type manufactured by Xilinx Corporation to realize functions of the four control loops. The PCB circuitry is shown in Figure 5(b). The gauge outfit of HRG is shown in Figure 5(a), the device of which has the shape of a cylinder with a diameter of 54 mm and a height of 72 mm. There is a prop-crib timbering outside the device which protects the device from damage.

The overall construction of the circuit is shown in Figure 5(c). Since the primary function of control loops were realized in the FPGA, the peripheral equipment is not so complex. The results of the measurements are transferred to the upper computer through a serial port in digital form.

The experiment was carried out under room temperature about 25 °C. Both the primary vibrating frequency and the secondary vibrating frequency were tuned to 4,433.403 Hz by the control loops. The Lissajous figure on two electrodes with angle distance by 45° under well-balanced working mode is shown in Figure 2(b).

## 5. Data Analysis and Results

**Figure 6.**
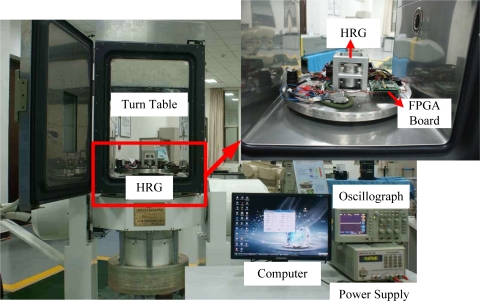
The experimental setup of the gyro.

In order to validate the effect of the digital control loops and the performance of the gyro, tests were performed by using a turntable as shown in Figure 6. The power and signals were transmitted through a slip ring to the related HRG device and an oscillograph was applied to monitor the working status of the HRG. Furthermore, digital output signal results were directly sent to the upper computer through a serial port and were stored in the hard disk for further study.

In order to obtain the scale factor of gyroscope, the turntable rotated from −9 to 9°/s respectively for 20 minutes. The data of the test results were recorded in the upper computer by a serial port with a baudrate of 921,600 bit/s and a frequency of 200 Hz. Then, by averaging the data obtained in each period as shown in Table 1 and by using the least squares fitting method, we get a straight line at last, which is expressed as:

(13) y=0.0238x−0.00062

**Figure 7.**
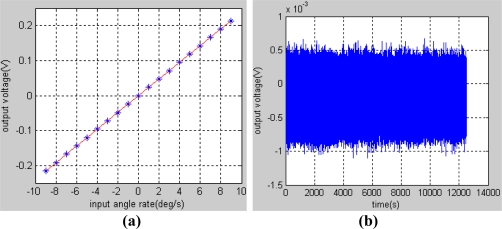
**(a)** The fitting curve of the test data. **(b)** Original data of gyroscope output in static state.

The fitting curve is shown in Figure 7(a) and it can be calculated that the scale factor of the gyroscope is 23.8 mV/°/s and the nonlinearity is 1.33% in the pre-set input range. An experiment was also carried out in static state on the marble foundation to get the bias stability of the gyroscope. The original data of gyroscope output is shown in Figure 7(b), whose sample frequency is 200 Hz. In order to evaluate the bias stability of the gyroscope, 2,000 original points of the data were averaged into one point, from which the standard variance can be deduced:

$$\mathrm{std}=3.8681\times 10^{-6}\left(V\right)$$

Then the bias stability can be calculated:

Bias Stability=std/0.0238(V/°/s)×3600(s)=0.5851°/h

## 6. Conclusions

A FPGA digital circuit board design for a HRG is realized based on the proposed control scheme and math model and relative experiments that are described in this paper. To begin with, the math model of the HRG with different kinds of errors on the resonator was theoretically studied. Secondly, control loops were employed to complete the manipulation of HRG with the errors suppressed or compensated. Lastly, experiments were carried out at room temperature to evaluate the digital design with a turntable and those studies reveal that the HRG has both a higher linearity scale factor and lower bias stability. Performance of the gyroscope suggests that the HRG would meet the needs of a wide range of commercial and military applications. In further research full temperature zone tests will be carried out and the temperature model of HRG will be established for drift compensation since the natural frequency of the resonator is closely related to the temperature.

## Figures and Tables

**Table 1.** The averaged value of each period.

Input angle rate (°/s)	The average output voltage (*V*)	Input angle rate (°/s)	The average output voltage (*V*)
−9	−0.214966	0	−0.000304
−8	−0.191252	1	0.023339
−7	−0.167513	2	0.047094
−6	−0.143742	3	0.070885
−5	−0.119948	4	0.094699
−4	−0.096113	5	0.118541
−3	−0.072255	6	0.142410
−2	−0.048329	7	0.166241
−1	−0.024335	8	0.190027
0	−0.000304	9	0.213734
